# Understanding the link between somatosensory temporal discrimination and movement execution in healthy subjects

**DOI:** 10.14814/phy2.12899

**Published:** 2016-09-20

**Authors:** Antonella Conte, Daniele Belvisi, Nicoletta Manzo, Matteo Bologna, Francesca Barone, Matteo Tartaglia, Neeraj Upadhyay, Alfredo Berardelli

**Affiliations:** ^1^ IRCCS NeuromedPozzilli (IS)Italy; ^2^ Department of Neurology and PsychiatrySapienza University RomeRomeItaly

**Keywords:** Movement execution, sensory gating, sensory temporal discrimination

## Abstract

The somatosensory temporal discrimination threshold (STDT) is the shortest interval at which an individual recognizes paired stimuli as separate in time. We investigated whether and how voluntary movement modulates STDT in healthy subjects. In 17 healthy participants, we tested STDT during voluntary index‐finger abductions at several time‐points after movement onset and during motor preparation. We then tested whether voluntary movement‐induced STDT changes were specific for the body segment moved, depended on movement kinematics, on the type of movement or on the intensity for delivering paired electrical stimuli for STDT. To understand the mechanisms underlying STDT modulation, we also tested STDT during motor imagery and after delivering repetitive transcranial magnetic stimulation to elicit excitability changes in the primary somatosensory cortex (S1). When tested on the moving hand at movement onset and up to 200 msec thereafter, STDT values increased from baseline, but during motor preparation remained unchanged. STDT values changed significantly during fast and slow index‐finger movements and also, though less, during passive index‐finger abductions, whereas during tonic index‐finger abductions they remained unchanged. STDT also remained unchanged when tested in body parts other than those engaged in movement and during imagined movement. Nor did testing STDT at increased intensity influence movement‐induced STDT changes. The cTBS‐induced S1 cortical changes left movement‐induced STDT changes unaffected. Our findings suggest that movement execution in healthy subjects may alter STDT processing.

## Introduction

Ample evidence describes changes in brain processing of tactile sensory information while healthy subjects prepare and execute voluntary movements (Brown et al. 2013). For example, early before and during motor execution, tactile sensitivity decreases owing to sensory gating (sensory attenuation) (Angel and Malenka 1982; Milne et al. 1988; Shergill et al. 2003). Motor execution also distorts tactile stimuli localization and duration in the spatial domain (Dassonville 1995), so that during internally generated motor actions tactile stimuli are mislocalized in the direction of the movement (Dassonville 1995; Watanabe et al. 2009; Maij et al. 2011). Movement also influences tactile information processing in other ways unrelated to suppression. When subjects executed goal‐directed movements, event‐related potentials at 80–200 msec after tactile stimulation and at 80–100 msec after visual stimulation were enhanced (Juravle et al. 2016). Overall these observations show that incoming sensory information is continuously monitored so as to adjust the current motor plan (Juravle et al. 2016). Motor execution also distorts tactile stimulus processing in the temporal domain (Yarrow et al. 2001; Haggard et al. 2002; Morrone et al. 2005; Hagura et al. 2012; Tomassini et al. 2012). For example, when subjects prepare and execute hand movements they perceive intervals marked by tactile stimuli as shorter than those unaccompanied by movements (Tomassini et al. 2012).

In humans, one way to assess tactile temporal processing is to calculate the somatosensory temporal discrimination threshold (STDT). The STDT is the shortest interval at which an individual recognizes paired stimuli as separate in time (Conte et al. 2010, 2012, 2014; Tinazzi et al. 2013; Rocchi et al. 2016). The STDT relies on a purely sensory process that allows the brain to filter out irrelevant sensory information coming from external sources (Conte et al. 2013). Whether and how voluntary movement modulates the STDT in healthy subjects is unknown. Having this information might help in designing studies to clarify the pathophysiological mechanisms underlying the altered STDT values reported in patients with movement disorders (Artieda et al. 1992; Bradley et al. 2009; Scontrini et al. 2009; Conte et al. 2010, 2014; Lee et al. 2010; Tinazzi et al. 2013; Kimmich et al. 2014).

We designed this study to investigate whether movement execution and preparation – motor functions involving motor circuit activation – influence STDT in healthy subjects. To do so, we first tested STDT during voluntary index finger abductions at several time points after movement onset and during motor preparation. We then tested whether the STDT changes induced by voluntary movement were specific for the body segment moved, depended on movement kinematics, or on the type of movement or on the intensity used for delivering paired electrical stimuli for STDT. To test whether the electrical stimuli we delivered for STDT brought about changes in movement kinematics and duration, we also recorded movements and analyzed their kinematic features. Finally, to understand the mechanisms underlying movement‐induced STDT changes, we tested STDT during motor imagery and after delivering repetitive transcranial magnetic stimulation to elicit excitability changes in the primary somatosensory cortex (S1).

## Methods

For the study, we enrolled 17 healthy subjects (aged 24–43 years) at the Department of Neurology and Psychiatry.

### Ethical approval

All participants gave written informed consent. The experimental procedure was approved by the institutional review board at Sapienza University Rome (CE REF# 4041) and conducted in accordance with the Declaration of Helsinki.

### STDT testing

During the study, participants were comfortably seated in an armchair beside a table. STDT was investigated according to the experimental procedures used in previous studies (Conte et al. 2010, 2012, 2014). We delivered paired stimuli starting with an interstimulus interval (ISI) of 0 msec (simultaneous pair), and progressively increased the ISI in 10‐msec steps. Paired tactile stimuli consisted of square‐wave electrical pulses delivered with a constant current stimulator (Digitimer DS7AH) through surface skin electrodes with the anode located 0.5 cm distally to the cathode. Stimulation intensity was defined for each subject by delivering a series of stimuli at increasing intensity from 2 mA in 0.5 mA steps; the intensity used for STD testing was the minimal intensity, the subject perceived in 10 out of 10 consecutive stimuli. The first of three consecutive ISIs at which participants recognized the stimuli as temporally separate was considered the STDT. To keep the subjects’ attention level constant during the test and minimize possible perseverative responses, we included “catch” trials consisting of a single stimulus delivered randomly.

### Movement recording and motor tasks

The SMART analyzer motion system (BTS Engineering, Milan, Italy), equipped with three infrared cameras (sampling rate, 120 Hz), was used to record index finger abductions and proximal arm movements. For index finger movements, the right arm was firmly secured and the arm position was kept constant throughout the experiment by visually inspecting the joint angles and by keeping the distance between the armchair and the table stable. Care was taken to secure the right forearm firmly in the same position on the table. The right arm was abducted at the shoulder by about 45–50° and the elbow joint was flexed at about 90° (Agostino et al. 2007). An optical marker was placed over the distal phalanx of the dominant index finger. After a verbal “go” signal subjects abducted the index finger and soon after a verbal “stop” signal they returned the finger to the starting position (Agostino et al. 2007; Li Voti et al. 2011, 2014; Bologna et al. 2015). For proximal arm movements, subjects were instructed to abduct the arm as fast as possible. The endpoint marker was placed on the right elbow. During deltoid muscle activation electromyographic (EMG) signal from the first dorsal interosseous (FDI) muscle was monitored constantly to ensure that hand muscles were kept relaxed. Marker displacement for all the motor tasks was reconstructed via dedicated software running the automatic algorithm to compute acceleration and velocity peak values (BTS Engineering, Milan, Italy).

### Recording techniques

#### Electromyographic recordings

Electromyographic activity was recorded through a pair of surface (Ag/AgCl) cup electrodes placed over the FDI muscle or the deltoid muscle, in a belly‐tendon configuration. EMG signals were recorded and filtered with a Digitimer D360 (Digitimer Ltd, UK) (bandwidth 20 Hz–1 kHz), then analyzed off‐line with a personal computer through a 1401 plus A/D laboratory interface (Cambridge Electronic Design, UK).

Data were stored on a laboratory computer for on‐line visual display and further off‐line analysis (Signal software; Cambridge Electronic Design).

### Experimental techniques

All 17 healthy subjects underwent the main experimental protocol first and some took part in the control experiments (applied in random order).

#### Index finger abductions and STDT tested on the right index finger (motor execution and motor preparation)

Paired stimuli for STDT were triggered by movement execution at various intervals after movement onset. The movement consisted in right index finger abductions and the STDT was tested on the volar surface of the right index finger. Subjects were asked to abduct the index finger as widely and as fast as possible and were continuously encouraged to do so throughout the motor task. To define the time course of movement‐induced STDT changes, paired stimuli were delivered at five intervals: as soon as the EMG signals reached 100 μV in amplitude (defined as “0 msec” for simplicity), 100, 200, 500 msec, and 5 sec after movement onset, and STDT values were calculated for each interval. Specifically, paired stimuli for STDT testing at the 0‐msec interval were delivered concomitantly with movement onset. The subject was asked to abduct the right index finger while paired electrical stimuli were delivered at 0 msec after each movement onset. The threshold to identify movement onset for fast as well as slow index finger abductions was set at 100 μV of EMG activity. At each index finger abduction, the interval for STDT testing was progressively increased in 10‐msec steps, until the subject recognized the two stimuli as sequential (Conte et al. 2012). The STDT was defined as the average of three STDT values, that is, one for each block and was entered in the data analysis. Similarly, paired stimuli for STDT testing in the four trials were delivered at 100‐, 200‐, 500‐msec and 5‐sec intervals after movement onset.

**Figure 1:**
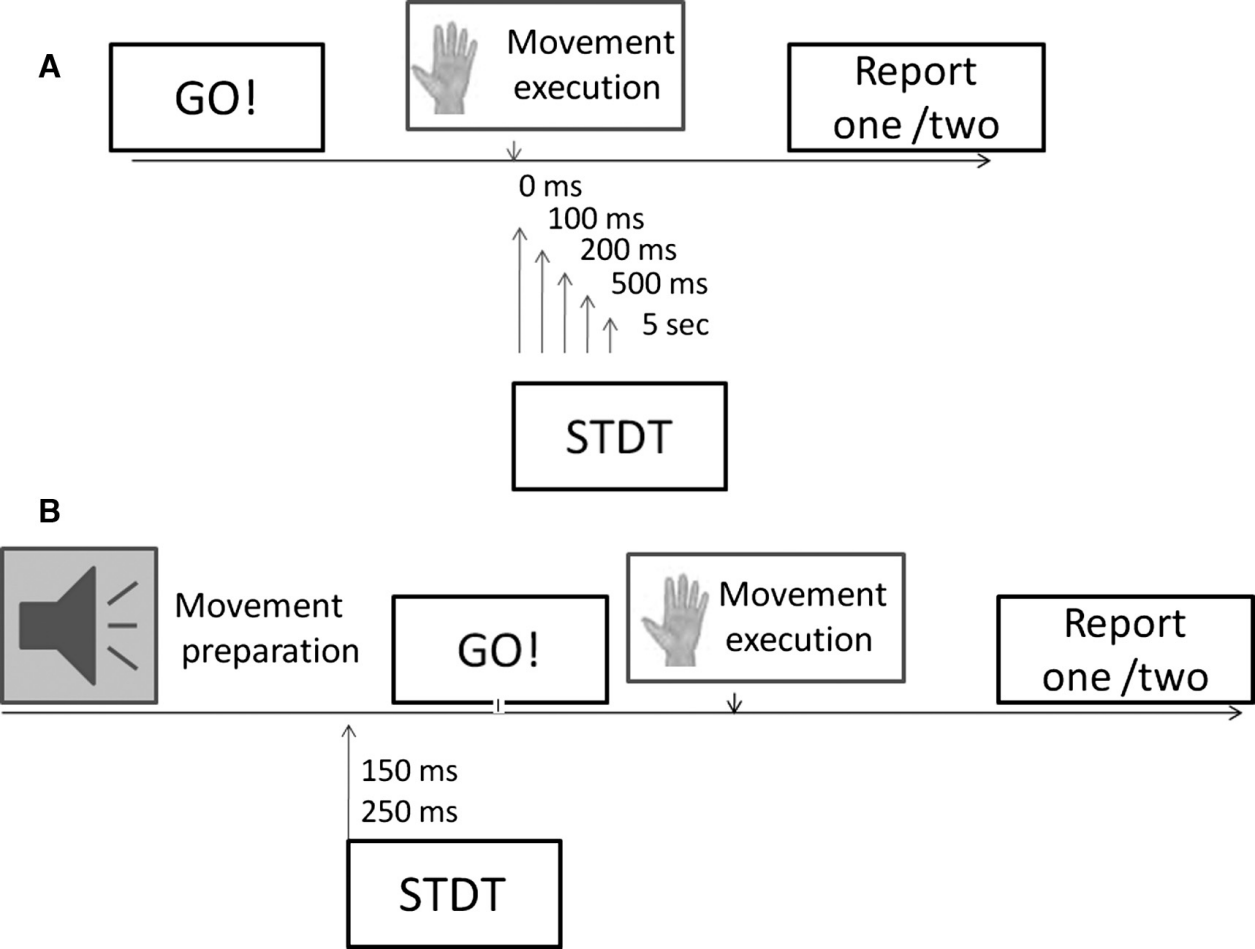
Experimental set‐up. (A) Somatosensory tactile discrimination threshold (STDT) values at movement onset and 100, 200, 500 msec and 5 sec thereafter. (B) STDT assessment during motor preparation. Paired electrical stimuli for STDT testing were delivered 250 msec and 150 msec before movement onset.

To evaluate STDT during motor preparation, after determining STDT values at baseline, we delivered paired stimuli for STDT between an acoustic signal indicating that the subject had to prepare the index finger movement and 250 msec and 150 msec before the “go” signal indicating that the subject had to start movement (Fig. 1, experimental setup). Trials were delivered in random order. From the EMG traces, we also measured the mean movement onset latency (EMG threshold of 100 μV) after the “Go” signal.

#### Topographic specificity of movement‐induced changes in STDT

To determine whether movement‐induced changes in STDT were specific for the body part moved, we ran three control experiments in 13 subjects. First, subjects were asked to abduct the right index finger while we delivered paired stimuli for STDT testing on the volar surface of the left index finger; they then had to abduct the right arm as fast as possible (deltoid muscle activation) while we tested STDT on the volar surface of the right index finger; and third, we asked them to abduct the right index finger and tested STDT on the volar surface of the right digiti minimi. Paired stimuli for STDT were delivered 0, 100, and 200 msec after movement onset.

#### Movement execution kinematics and STDT

To find out whether movement‐induced changes in STDT depended on movement velocity, in the same subgroup of 13 participants, we asked subjects first to abduct the index finger as slowly as possible. Paired stimuli for testing right‐finger STDT were then delivered at 0, 100 and 200 msec thereafter. In the second experiment, the investigator passively moved the index finger and paired stimuli for STDT were delivered concomitantly with movement onset (“0 msec”).

#### Tonic index finger abduction and STDT

To find out whether the STDT changes depended on the muscle contraction itself or on the type of movement (phasic, as described in the main experiment, vs. tonic), we asked 10 subjects to abduct the right index finger and keep the first dorsal interosseous muscle in a maximal tonic contraction with the aid of visual EMG feedback, while we delivered paired electrical stimuli for STDT to the right index finger.

#### Index finger abductions and STDT tested at increased intensity on the right index finger

In this experiment, in 13 participants, electrical stimuli for STDT were delivered at twice the intensity used in the main experiment at 0, 100, and 200 msec after the right index finger abductions.

#### Role of cortical areas in the movement‐induced STDT changes

To gain further information on the cortical areas responsible for STDT modulation, we investigated STDT values during motor imagery while participants mentally executed index finger abductions. Paired electrical stimuli for STDT testing were delivered to the right index finger while the subjects imagined they were abducting the index finger. A “go” signal was used to trigger the paired electrical stimuli for STDT testing and to tell subjects when to begin the imagined right index finger abductions.

To investigate whether movement‐induced changes in STDT take place in the S1 cortex, in 10 subjects, we applied repetitive transcranial magnetic stimulation over the primary sensory cortex (S1), according to the experimental neurophysiological technique continuous theta‐burst stimulation (cTBS) (Huang et al. 2007). Because cTBS decreases S1 cortical excitability (Ishikawa et al. 2007; Conte et al. 2012) for about 20 min after stimulation ends, we evaluated STDT during movement execution (“0 msec” experiment) before and 10 min after cTBS over S1. Intensity for cTBS was set at 80% active motor threshold (AMT). We stimulated S1 at the site described in previous studies (Okamoto et al. 2004; Wolters et al. 2005; Ishikawa et al. 2007), a point 2 cm posterior to M1 right‐hand muscle motor hotspot determined by single pulse TMS. This point overlies the postcentral gyrus (Okamoto et al. 2004) and the rTMS over this position elicits after‐effects on the cortical somatosensory‐evoked potential (SEP) component (Ishikawa et al. 2007). Percent changes in STDT values (STDT at “0 msec”/STDT baseline) were calculated before and after S1 cTBS and the ratio was entered in the data analysis.

### Statistical analysis

Unless otherwise stated, all values are means ± standard error (SE). To analyze changes in STDT values during index finger abductions in the main experiment, we used a repeated measures analysis of variance (ANOVA) with factor ISI (ISI: 6 levels: baseline, 0, 100, 200, 500 msec, and 5 sec after movement). To analyze whether STDT values changed before the index finger movement (motor preparation), we used a repeated measures ANOVA with factor ISI (3 levels: baseline, 250 msec and 150 msec before index finger movement). To analyze changes in STDT values in control experiments, we ran a repeated measures ANOVA with factors EXPERIMENTAL SESSION (five levels) and ISI (4 levels: baseline, 0, 100, 200 msec after movement onset). To analyze changes in movement variables for right index finger abductions within and across different experimental sessions, we used a further ANOVA with factor EXPERIMENTAL SESSION (two levels: when STDT was tested on the right and left hands) and ISI (three levels: 0, 100, and 200 msec after movement onset). The Greenhouse‐Geisser correction was applied when needed. Tukey's test was used for post hoc analysis. To analyze whether STDT values changed during motor imagery, during passive index finger abductions, during muscle tonic contraction, and after S1 cTBS (STDT percentage change at 0 msec pre‐S1 cTBS vs. STDT percentage change at 0 msec post‐S1 cTBS), we used a paired sample *T* test. Pearson's correlation coefficient was used to disclose any relations between the STDT changes (expressed as STDT at “0 msec”, “100 msec”/baseline STDT ratio) and index finger abduction (peak acceleration and peak velocity) kinematics. *P* values <0.05 were considered to indicate statistical significance.

## Results

### Index finger abductions and STDT tested on the right index finger (motor execution and motor preparation)

**Figure 2:**
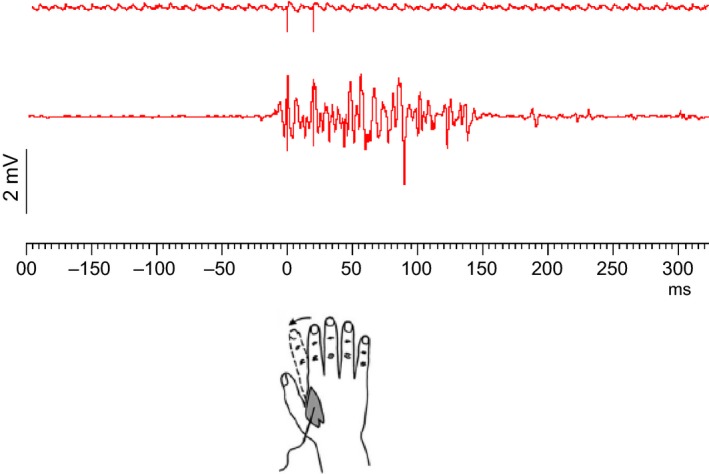
Electromyographic (EMG) trace for fast index finger abduction and timeline of paired electrical stimuli for somatosensory temporal discrimination threshold (STDT) in a representative subject. Figure represents the “0 msec” condition. The artifacts related to the paired electrical stimuli for STDT (interstimulus interval: 20 msec) are shown in the upper panel. The lower panel shows EMG activity recorded through surface electrodes placed over the first dorsal interosseous muscle corresponding to the forward movement in the fast index finger abduction.

**Figure 3:**
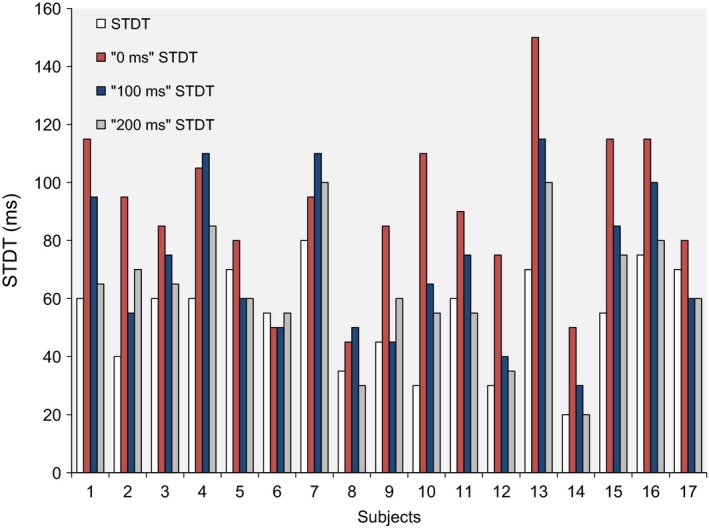
Somatosensory temporal discrimination threshold (STDT) values at baseline, at movement onset (0 msec) and 100, 200 msec after movement onset in the 17 healthy subjects who took part in the main experiment.

**Figure 4:**
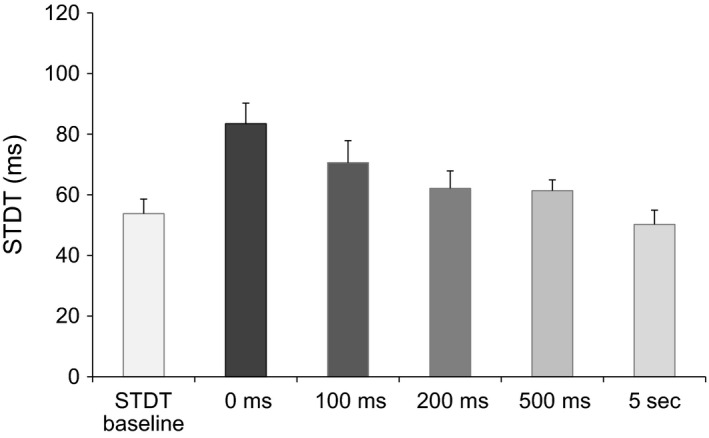
Somatosensory temporal discrimination threshold (STDT) values at movement onset and 100, 200, 500 msec and 5 sec thereafter. Histograms represent STDT values at the different time points. Grayscale suggests different statistical significance, higher at 0 and 100 msec intervals.

Repeated measures ANOVA showed a significant factor ISI (*F*_5,80_ = 19.36, *P* < 0.000001). Post hoc tests showed that STDT values increased significantly when paired stimuli were delivered after the onset of right index finger abductions: at 0 msec (*P* = 0.0001), 100 msec (*P* = 0.002) and 200 msec (*P* = 0.04). STDT values returned to baseline values at 500 msec (*P* = 0.6) and 5 sec (*P* = 0.15) after movement onset (Figs. 2, 3, 4). No significant correlation was found between changes in the STDT values expressed as STDT percentage increase at 0 and 100 msec in comparison with baseline values (0 msec: 185 ± 15%, 100 msec: 139 ± 8%) and finger abduction kinematics (EMG duration: 323 ± 43 msec, *P* = 0.33; peak velocity: 334 ± 31 mm/sec: *P* = 0.44 and *P* = 0.68; peak acceleration: 11.3 ± 1 mm/sec^2^; *P* = 0.75 and *P* = 0.34). Peak velocity of index finger‐abduction remained significantly unchanged across the various conditions testing different intervals elapsing after movement onset and paired stimuli for STDT (*F* = 1.68, *P* = 0.15). Nor did movement duration and kinematics of right index finger abductions vary when the STDT was tested on the left hand (EMG duration: factor EXPERIMENTAL SESSION *F* = 0.98, *P* = 0.37 and factor ISI *F* = 0.18, *P* = 0.83; peak acceleration: factor EXPERIMENTAL SESSION *F* = 0.45, *P* = 0.53 and factor ISI *F* = 0.06, *P* = 0.94; peak velocity: factor EXPERIMENTAL SESSION *F* = 3.2, *P* = 0.1 and factor ISI *F* = 0.36, *P* = 0.69).

Repeated‐measures ANOVA for STDT changes during motor preparation showed no significant factor ISI (*F*_2,32_ = 1.44, *P* = 0.26). STDT values therefore remained statistically unchanged during motor preparation at both 250 msec and 150 msec before movement onset (STDT at baseline: 54.6 ± 4 msec; STDT at 250 msec before movement onset: 55.7 ± 4 msec; STDT at 150 msec before movement onset: 60.8 ± 3 msec). Mean movement onset latency was 219 ± 12 msec.

### Topographic specificity of STDT modulation induced by movement execution

**Figure 5:**
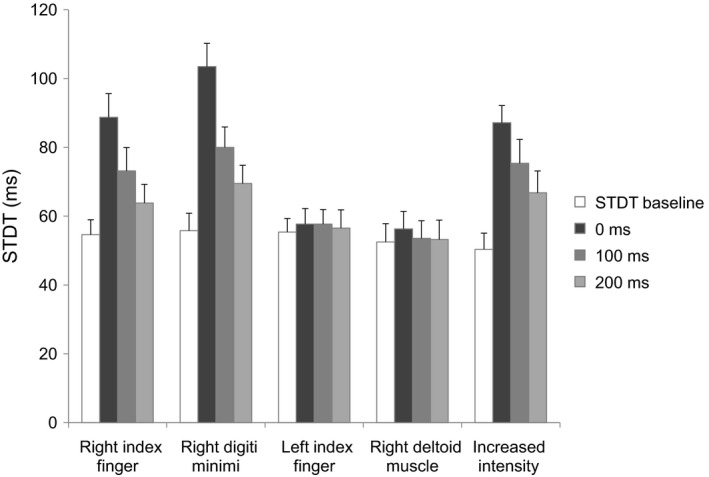
Somatosensory temporal discrimination threshold (STDT) tested on the right index finger during right index finger abduction, on the right digiti minimi during index finger abduction, on the left index finger during right index finger abduction, on the right index finger during ipsilateral deltoid muscle abduction and STDT tested at increased intensity on the right index finger during index finger abduction. STDT values were tested at movement onset and at 100 and 200 msec thereafter.

Repeated measures ANOVA showed a significant factor EXPERIMENTAL SESSION (*F*_5,48_ = 9.71, *P* < 0.00003), ISI (*F*_3,36_ = 44.2, *P* < 0.000001) and a significant EXPERIMENTAL SESSION × ISI interaction (*F*_12,144_ = 8.60, *P* < 0.0000001). Post hoc analysis showed that in the main experiment, changes in STDT values were significant after the onset of right index finger abductions (*F*_3,36_ = 19.31, *P* < 0.00001) (at the 0‐msec ISI, *P* = 0.00001, 100‐msec ISI, *P* = 0.001 and 200‐msec ISI, *P* = 0.02), and in the control experiments when STDT was tested on the right digiti minimi, after the onset of right index finger abductions (*F*_3,36_ = 33.7, *P* < 0.0001) (at the 0‐msec ISI, *P* = 0.00015, 100‐msec ISI, *P* = 0.0002 and 200 msec ISI, *P* = 0.03). Conversely, when STDT was tested on the left‐index finger during right index finger abductions STDT values remained unchanged (*F*_3,36_ = 4.44, *P* = 0.72) and also when tested on the right index finger during proximal arm movements (*F*_3,36_ = 1.60, *P* = 0.22) (Fig. 5).

### Kinematic properties of movement execution and STDT

**Figure 6:**
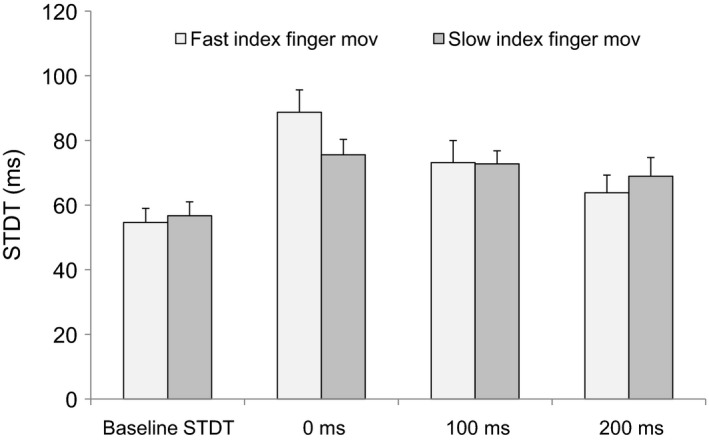
Somatosensory temporal discrimination threshold (STDT) during fast as well as slow index finger abductions. STDT values were tested at movement onset and at 100 and 200 msec thereafter.

When subjects did slow index finger abductions (EMG duration: 726 ± 43 msec; peak velocity: 127 ± 33 mm/sec; peak acceleration: 4.3 ± 0.3 mm/sec^2^) STDT values changed significantly at movement onset and within 200 msec thereafter (*F*_3,36_ = 6.75; *P* = 0.0001; 0 msec: *P* = 0.0007; 100 msec: *P* = 0.02; 200 msec: *P* = 0.02) (Fig. 6).

Paired sample *T* test showed that STDT increased during passive movement (*P* = 0.01) although it increased less than it did during voluntary index finger abductions.

### Tonic index finger abduction and STDT

Paired *T* test showed no significant changes in the STDT values tested during first dorsal interosseous muscle tonic contractions (STDT at baseline: 56.2 ± 6 msec vs. 54.3 ± 7 msec during tonic contraction; *P* = 0.56).

### Index finger abductions and STDT tested at increased intensity on the right index finger

Somatosensory temporal discrimination threshold changes were also significant when STDT values were tested at two‐fold higher intensities (*F*_3,36_ = 27.28, *P* < 0.0001) at 0 msec (*P* = 0.000164), 100 msec (*P* = 0.000166) and 200 msec (*P* = 0.002) after right index finger abductions (Fig. 5). None of the subjects reported stimulation intensity as painful.

### Role of cortical areas in the movement‐induced STDT changes

Paired sample *T* test showed no significant changes in STDT during the motor imagery task (STDT at baseline: 53.3 ± 6 msec vs. STDT during motor imagery: 53.8 ± 7 msec; *P* = 0.82).

Primary somatosensory cortex cTBS significantly increased baseline STDT values but left percentage STDT change at movement onset unmodified (baseline STDT values before S1 cTBS: 57 ± 3 msec vs. baseline STDT values after S1 cTBS: 69 ± 3 msec; *P* < 0.0001; percentage change at 0 pre‐S1 cTBS vs. STDT percentage change at 0 msec post‐S1 cTBS: 149 ± 9% vs. 146 ± 11%; *T* test: *P* = 0.48).

## Discussion

In this study, in healthy subjects we now provide new evidence showing that movement execution brings about changes in the temporal processing of tactile information – as tested with STDT. When we tested STDT on the moving hand (index and digiti minimi fingers) at movement onset and up to 200 msec thereafter, STDT values increased from baseline. Conversely, during motor preparation, STDT remained unchanged. We also provide new observations showing how movement execution modulates STDT values: in all our healthy subjects, STDT values changed significantly during fast as well as slow index finger movements and, although to a lesser extent, also during passive index finger abductions, whereas during tonic index finger abduction they remained statistically unchanged. No differences were found in STDT when tested in body parts other than those involved in movement execution and during imagined movement. Nor did increasing the intensity used for STDT influence movement‐induced changes in STDT. Changes in S1 cortical activity with cTBS increased significantly STDT values but left the movement‐induced STDT changes unaffected. Overall, we show that movement‐related STDT values become evident only when STDT is tested on the same body part engaged in the motor task thus giving further insight into altered STDT in movement disorders.

Our experimental procedures envisaged several precautions to avoid bias in interpreting our findings. To exclude attentional‐related changes in STDT, we checked possible changes in subjects’ attention levels by delivering “catch trials”. The single stimulus used to check possible changes in attentional levels when the ISI was higher than that corresponding to the threshold excluded a perseverative response. We also took into account possible changes in attention levels, given that we measured STDT three times and then calculated the mean of the three values. The low standard deviation in single subjects provided information on participant performance reliability (average standard deviation for the three STDT values: 5.3 msec‐*data not shown*). Further evidence confirming participants’ performance reliability came from our observation that baseline STDT values remained statistically unchanged across the various experimental sessions. To make sure that fatigue or distraction did not distort the trial results, we gave experiments in randomized order. Our experiments excluded aspecific movement‐induced STDT changes attributable to “dual task effects” (a reduced discriminative ability owing to the two contemporary tasks), because the STDT tested on the hand contralateral to that involved in movement remained unchanged during and after the movement. This conclusion receives support also from our experiments showing that the three tasks testing movement preparation caused no significant STDT changes. Because movement‐induced STDT changes also when we delivered stimuli at an intensity twice higher than that used in the main experimental procedure, we exclude STDT changes depending entirely on sensory attenuation mechanisms (Chapman et al. 1987; Roussel et al. 2014).

Our observation that the STDT undergoes movement‐related changes when this variable is tested on the same body part engaged in the voluntary motor task suggests sensory task‐related changes in the temporal domain. Hence it concurs with evidence that somatosensory‐evoked potentials from the primary somatosensory cortex (S1) decrease in amplitude during movement execution (Chapman et al. 1987). Ample evidence describes SEP attenuation (Coquery 1971; Chapman et al. 1987) during movement execution as well as during motor preparation (Angel and Malenka 1982; Milne et al. 1988; Shergill et al. 2003; Seki and Fetz 2012) probably because SEP is centrally gated. Similarly, in experiments using SEP to investigate sensory gating in monkeys, Seki and Fetz (2012) also suggested that sensory input is gated in the S1 cortex. Our findings might therefore imply that STDT modulation takes place through gating processes in the S1 cortex or through M1‐S1 cortico‐cortical connections. We and others previously showed that cTBS decreases S1 cortical excitability (Ishikawa et al. 2007; Conte et al. 2012) for about 20 min after stimulation ends. If cTBS over S1 in our experiments had changed the extent to which movement execution modulated STDT, then STDT modulation presumably takes place in the S1 cortex. Conversely, although S1 cTBS significantly increased baseline STDT values, thus implying that cTBS modulated S1 activity, it left the percentage movement‐induced changes in STDT unmodified. Hence STDT modulation in an S1 cortical site seems unlikely. Similarly, we tentatively consider it unlikely that M1 activation during index finger abductions inhibits sensory temporal processing in S1 through cortico‐cortical M1‐S1 pathways, given that STDT remained unchanged during motor preparation and also during the imagined motor action. Numerous studies conducted with functional MRI (fMRI) and a review show activity in cortical areas – motor cortices, posterior parietal cortex and SMA – during a motor imagery task (Lacourse et al. 2005; Hétu et al. 2013; Blefari et al. 2015; Ridderinkhof and Brass 2015; Pilgramm et al. 2016). Hence if STDT modulation during movement execution takes place at the cortical level, mental imagery of the index finger abductions should at least partly influence STDT values. Even though mental imagery may also recruit subcortical structures, a recent study with fMRI showed that mental imagery activates the head of the caudate, a brain structure belonging to the associative basal ganglia circuitry (Tremblay et al. 2015). Sensorimotor processes, such as those we investigated, mostly involve the putamen. Our findings during imagined movement therefore seem to argue against STDT modulation originating in the cortex. They also suggest that associative circuits contribute little or nothing to the STDT. Although STDT depends on the activity in inhibitory interneurons in S1, some evidence suggests that the secondary somatosensory cortex (SII) has a role in integrating subsequent somatosensory input (Romo et al. 2002). Although STDT modulation might therefore take place in SII, current knowledge pertains to discriminative tasks involving working memory. In our study, to avoid STDT changes related to working memory and to previous perceptive experience, we specifically asked subjects to report what they perceived for each trial as it was presented. Studies conducted in recent years suggest that temporal processing involving short ISIs is a task requiring highly perceptual discrimination not accessible to cognitive control (Koch et al. 2009). Overall, we therefore consider an SII role in STDT processing unlikely. Finally, arguing against STDT modulation originating in the cortex, we found no correlation between changes in STDT values and the kinematic variable movement velocity, STDT undergoing changes during fast as well as slow index finger abductions. Our experimental design envisaged an interaction between a descending motor output underlying index finger movements and the ascending sensory information related to STDT. The concept that STDT is encoded at the cortical level in the primary or partly in the secondary somatosensory cortex or both allows for the possibility that sensory information related to the STDT might be gated elsewhere. Ample evidence in the literature, specifically animal studies, demonstrated that sensory gating takes place at different levels of the nervous system. Hence an alternative hypothesis is that STDT modulation depends on gating processes taking place in subcortical areas. “Subcortical” structures putatively involved in sensory gating include the spinal cord. Several animal studies suggested that spinal gating may suppress sensory signaling in a nonspecific way (Seki and Fetz 2012). During active movement, responses from first‐order spinal sensory interneurons decrease owing to presynaptic inhibition exerted by descending motor commands and are also collaterally inhibited by re‐afferent proprioceptive activity (Li et al. 2002; Seki and Fetz 2012). Despite these observations on spinal sensory gating, spinal gating seems unlikely to explain STDT modulation in healthy subjects we studied, especially insofar as the ipsilateral arm abduction (deltoid muscle‐receiving C5‐C6 innervation) left STDT tested on the index finger (C6 dermatome) unaffected. Nor did tonic FDI muscle activation influence STDT values thus again excluding STDT modulation related to presynaptic inhibitory mechanisms. Finally, during the phasic index finger abduction, up to 200 msec after movement onset STDT values increased, returning to baseline levels only at 500 msec after movement onset, when the index finger abduction was still ongoing. The foregoing observations also exclude STDT modulation related simply to the muscle contraction per se. Overall, our results also argue against STDT modulation predominantly reflecting reduced first‐order sensory transmission in the spinal cord.

Conversely, as a more likely explanation and one meriting future research, we suggest that STDT modulation during movement execution relies mainly on basal ganglia–thalamus interplay. A recent study in monkeys (Song and Francis 2015) demonstrated that tactile stimulation evokes oscillatory activities across the sensorimotor loop, with a strong directional coupling between the ventral posterolateral (VPL) nucleus of thalamus and S1. Temporal coding or synchrony seems important for regulating sensory input in the VPL. Upper‐limb muscle activation regulates neural oscillatory activities differentially, preferentially suppressing VPL‐S1 functional connections in the ascending direction (Song and Francis 2015). Once movement is initiated in M1, motor command flows from M1 through the basal ganglia thus facilitating pathways involved in the selected movement and inhibiting competing actions (Mink 1996; Redgrave et al. 1999; Colder 2015). According to the “re‐afference principle” (Von Holst and Mittelstaedt 1950) copies of motor commands are transmitted to sensory processing regions so that action is tied to selected sensory expectation (Colder 2015). Information flows from the cortex through the basal ganglia and thalamus back to the cortex (Alexander et al. 1986; Middleton and Strick 2002; Haber and Calzavara 2009), the thalamus receiving massive topographically organized projections from the basal ganglia (specifically the striatum) (Haber and Calzavara 2009). Previous recordings in animals showed that limb movement elicits various types of neuronal activity (Mink 1996). One neural activation pattern is somato‐topically organized, time‐locked to movement, and is seen when specific sensory stimuli, especially those presented during movement, cause neuronal firing. About 40% of movement‐related putamen neurons also responded to somatosensory stimuli even during passive movements (Crutcher and DeLong 1984; Mink 1996). This observation is in line with our finding that passive movement caused a mild increase in STDT values. In this view, we hypothesize that basal ganglia activation at movement onset and during the initial movement execution stages may gate ascending STDT‐related inflow from the ventral‐posterolateral nucleus to S1 in favor of the expected resulting sensation associated with action, thus increasing the time the subject needs for tactile temporal discrimination. Sensory gating of tactile input may therefore take place in the initial movement stage to prioritize proprioceptive information, and as long as the movement goes on, other motor control circuits intervene. The indirect evidence we provide suggesting basal ganglia involvement in STDT modulation during movement execution deserves research designed to seek direct evidence from studies in patients.

In conclusion, our findings show that movement execution in healthy subjects interferes with STDT processing in subcortical areas, possibly through basal ganglia–thalamus connectivity thus linking STDT and motor circuits. Future studies in patients with movement disorders will provide insight into the STDT abnormalities reported in Parkinson's disease and dystonia (Conte et al. 2010, 2014; Kimmich et al. 2014).

## Conflict of Interest

None declared.
